# When is informed consent required in cluster randomized trials in health research?

**DOI:** 10.1186/1745-6215-12-202

**Published:** 2011-09-09

**Authors:** Andrew D McRae, Charles Weijer, Ariella Binik, Jeremy M Grimshaw, Robert Boruch, Jamie C Brehaut, Allan Donner, Martin P Eccles, Raphael Saginur, Angela White, Monica Taljaard

**Affiliations:** 1 Department of Epidemiology and Biostatistics, University of Western Ontario, London, ON, N6A 5C1, Canada; 2 Division of Emergency Medicine, University of Calgary, Foothills Medical Centre, 1403 29th Street NW, Calgary, AB, T2N 2T9, Canada; 3 Rotman Institute of Philosophy, Department of Philosophy, University of Western Ontario, London, ON, N6A 5B8, Canada; 4 Department of Medicine, University of Western Ontario, 339 Windermere Road, London, ON, N6A 5A5, Canada; 5 Ottawa Hospital Research Institute, Clinical Epidemiology Program, Civic Campus, 1053 Carling Avenue, Ottawa, ON, K1Y 4E9, Canada; 6 Department of Medicine, Faculty of Medicine, University of Ottawa, Ottawa, ON, K1H 8L6, Canada; 7 Graduate School of Education and Statistics Department, Wharton School, University of Pennsylvania, 3700 Walnut Street, Philadelphia, PA, 19104, USA; 8 Department of Epidemiology and Community Medicine, University of Ottawa, Ottawa, ON, K1H 8M5, Canada; 9 Robarts Clinical Trials, Robarts Research Institute, London, ON, N6A 5K8, Canada; 10 Institute of Health and Society, Newcastle University, Baddiley-Clark Building, Richardson Road, Newcastle upon Tyne, NE2 4AX, UK; 11 Department of Medicine, University of Ottawa and Ottawa Hospital, Ottawa Hospital Research Institute, 1053 Carling Avenue, Ottawa, ON, K1Y 4E9, Canada

## Abstract

This article is part of a series of papers examining ethical issues in cluster randomized trials (CRTs) in health research. In the introductory paper in this series, we set out six areas of inquiry that must be addressed if the cluster trial is to be set on a firm ethical foundation. This paper addresses the second of the questions posed, namely, from whom, when, and how must informed consent be obtained in CRTs in health research? The ethical principle of respect for persons implies that researchers are generally obligated to obtain the informed consent of research subjects. Aspects of CRT design, including cluster randomization, cluster level interventions, and cluster size, present challenges to obtaining informed consent. Here we address five questions related to consent and CRTs: How can a study proceed if informed consent is not possible? Is consent to randomization always required? What information must be disclosed to potential subjects if their cluster has already been randomized? Is passive consent a valid substitute for informed consent? Do health professionals have a moral obligation to participate as subjects in CRTs designed to improve professional practice?

We set out a framework based on the moral foundations of informed consent and international regulatory provisions to address each of these questions. First, when informed consent is not possible, a study may proceed if a research ethics committee is satisfied that conditions for a waiver of consent are satisfied. Second, informed consent to randomization may not be required if it is not possible to approach subjects at the time of randomization. Third, when potential subjects are approached after cluster randomization, they must be provided with a detailed description of the interventions in the trial arm to which their cluster has been randomized; detailed information on interventions in other trial arms need not be provided. Fourth, while passive consent may serve a variety of practical ends, it is not a substitute for valid informed consent. Fifth, while health professionals may have a moral obligation to participate as subjects in research, this does not diminish the necessity of informed consent to study participation.

## Introduction

This article is part of a series of papers examining ethical issues in cluster randomized trials (CRTs) in health research. CRTs are used increasingly in knowledge translation research, quality improvement research, community based intervention studies, public health research, and research in developing countries. While a small and growing literature explores ethical aspects of CRTs, cluster trials raise difficult issues that have not been addressed adequately. In the introductory paper in this series, we set out six areas of inquiry that must be addressed if the cluster trial is to be set on a firm ethical foundation [1]. These include identifying research subjects, obtaining informed consent, the applicability of clinical equipoise, benefit-harm analysis, the protection of vulnerable populations, and the role and authority of gatekeepers in CRTs. This paper addresses the second of the questions posed, namely, from whom, when, and how must informed consent be obtained in CRTs in health research?

Informed consent is the subject of discussion in the CRT literature. Edwards and colleagues describe the difficulties associated with obtaining informed consent in CRTs [2]. They distinguish between individual-cluster trials and cluster-cluster trials, and argue that the potential to obtain informed consent is linked to the "level" at which study interventions are administered. In individual-cluster trials, study interventions -- such as a patient-centered educational brochure or vaccination -- are directed at individual cluster members. These interventions could be studied in an individually randomized trial, but a CRT may be performed due to countervailing considerations of treatment contamination, a desire to study group effects, logistical demands, or other practical reasons [3]. Since the "individuals within clusters are given treatments, they can in theory consent individually to the treatment(s) offered within their cluster" [2]. Thus, generally, informed consent should be obtained in individual-cluster trials.

Cluster-cluster trials, on the other hand, evaluate experimental interventions -- such as mass education or a training program for health care providers -- that are applied to entire clusters [2]. In these studies, it may not be possible to obtain informed consent from individual cluster members. Since the study intervention is delivered at the cluster level, it may be very difficult for cluster members to avoid the intervention if they don't wish to participate in the CRT. In these cases, "individuals cannot act independently" and "the autonomy principle is lost" [2]. Further, when dealing with large clusters, it may be logistically difficult to obtain informed consent from all cluster members [2]. Thus, in cluster-cluster trials it may not be meaningful or feasible to obtain informed consent.

The United Kingdom Medical Research Council *Cluster Randomised Trials: Methodological and Ethical Considerations *("MRC guidelines") provides important guidance to researchers and research ethics committees on informed consent and other aspects of CRTs [4]. Broadly, the MRC guidelines follow the conclusions of Edwards and colleagues regarding informed consent. Individual level interventions generally "do allow individual choice" while cluster level interventions "do not allow individual choice" [4]. An important innovation of the document is the recognition that a single trial may contain both individual and cluster level interventions. Based on this insight, the authors conclude that informed consent should be sought where possible:

"The fact that individual choice does not exist for a [cluster level] intervention (or for cluster randomization) does not, for instance, prevent individual consent being sought for giving a complementary [individual level] intervention which is part of the intervention package, or for taking samples, recording information, or extracting data from records" [4].

They also suggest that when informed consent cannot be obtained for all aspects of the study, agreement to enroll a cluster in the study must be sought from a gatekeeper or "cluster representation mechanism" -- a person or body charged with making decisions on behalf of the entire cluster [4]. (The role and authority of gatekeepers in CRTs is the subject of another paper in this series).

These conclusions regarding the role of informed consent in CRTs seem -- for the most part -- intuitively correct. Moreover, they are reflected in practice: investigators routinely obtain informed consent in CRTs evaluating individual level interventions, and they often do not obtain informed consent in CRTs evaluating cluster level interventions [5,6]. Nonetheless, we believe that further work is required, since some ethical questions posed in the literature have resisted a conclusive resolution, and the literature on CRTs remains somewhat disconnected from that on research ethics. For conclusions regarding informed consent and CRTs to be deemed broadly convincing by researchers, research ethics committees, regulators, and funding agencies, they must be based explicitly on ethical principles and research regulations.

This paper seeks to answer the question of from whom, when, and how must informed consent be obtained in CRTs in health research? After reviewing the implications of prior work in this series of articles for informed consent, we present a conceptual foundation for informed consent based on ethical theory and the moral purpose of consent. We use this conceptual foundation and national and international regulatory provisions to address key questions related to informed consent in CRTs. First, how can a study proceed if informed consent is not possible? Second, is consent to randomization always required? Third, what information must be disclosed to potential subjects if their cluster has already been randomized? Fourth, is passive consent a valid substitute for informed consent? Fifth, do health professionals have a moral obligation to participate as subjects in CRTs designed to improve professional practice?

## Prior work

Prior work in this series of articles has implications for informed consent in CRTs in health research. In a previous paper we developed a novel definition of human research subject [7]. We argued that a human research subject is an individual whose interests may be compromised as a result of interventions in a research study [7]. This includes any person who is directly intervened upon by an investigator, who is deliberately intervened upon via manipulation of the environment, with whom an investigator interacts for the purpose of collecting data, or about whom an investigator obtains identifiable private information [7]. As a general rule, informed consent for study participation must be obtained from research subjects or their surrogate decision makers. There is no ethical or regulatory obligation to obtain informed consent (for research purposes) from people who do not meet the definition of human research subjects.

This understanding of human research subject has clear implications for informed consent in CRTs in health research. Consider a CRT conducted by Althabe and colleagues (the "Guidelines Trial") that used a behavioral intervention directed at health care providers to improve obstetrical care in Argentina and Uruguay [8]. The Guidelines Trial sought to develop and implement clinical guidelines for the use of episiotomy and the management of the third stage of labor. The primary outcome measures were the rates of episiotomy and prophylactic use of oxytocin in the third stage of labor. In total, 10 hospitals were randomized to the behavioral intervention, while 9 hospitals were randomized to receive no intervention. At each of the intervention hospitals, teams of 3 to 6 birth attendants (physicians, residents, and midwives) were identified as opinion leaders and they participated in a workshop to develop and disseminate clinical guidelines on the outcomes of interest. The teams then returned to their respective hospitals, disseminated guidelines, trained the other birth attendants, and implemented a system of reminders. Data on births were collected using a standard clinical record form, and no information identifying individual patients was transmitted from the hospitals. Outcome measures were collected at baseline, at the end of the 18 month intervention period, and 12 months thereafter. The study was approved by research ethics committees in Argentina, Uruguay, and the United States. Informed consent was obtained from all birth attendants in the intervention arm, and a waiver of consent for the patients in the study was obtained. The Guidelines Trial intervention resulted in a 68% absolute increase in the use of prophylactic oxytocin and an 11% absolute reduction in the use of episiotomy. These results remained stable 12 months after the end of the intervention period.

Who are the human research subjects in the Guidelines Trial and from whom is informed consent required? The birth attendants in hospitals allocated to the intervention arm of the study were directly intervened upon through training sessions and reminders, and accordingly they are human research subjects. Birth attendants in hospitals allocated to the control arm of the study were not intervened upon and hence they are not research subjects. Provided that it was feasible to do so, informed consent ought to have been obtained from birth attendants in the intervention arm of the study (indeed, their informed consent was obtained). Knowledge translation trials commonly intervene on health professionals to change their practice behaviours. An important implication of our prior work is that, in these cases, health providers are research subjects and there is a presumption that their informed consent is required [7].

The patients in the Guidelines Trial (whether in the intervention or the control arm) were not in a position whereby their interests could plausibly be compromised in the context of a research study and, accordingly, they were not human research subjects. The study intervention sought to bring the behaviour of birth attendants with regard to the use of episiotomy and the management of the third stage of labour in line with the best available medical evidence. (Of course, it was not known at the beginning of the study whether the study intervention would be effective in doing so.) Importantly, the fiduciary relationship between birth attendants and patients remained intact and, as a result, birth attendants were unimpeded in their ability to make medical decisions in the best interests of their patients. With regard to the specific criteria listed above, patients in the Guidelines Trial were not directly intervened upon by an investigator, they were not deliberately intervened upon via manipulation of their environment, and investigators did not interact with them for the purpose of collecting data. Data were collected on standard clinical forms and no identifying information on patients was transmitted beyond the hospital. Since the patients in the Guidelines Trial were not human research subjects, there was no obligation to obtain their informed consent. As a result, it was unnecessary to secure a waiver of consent for the study. Thus, another important implication of our prior work is that patients in knowledge translation trials who are merely indirectly impacted by study interventions are not human research subjects, and their informed consent is not required.

## A moral foundation of informed consent

In the introductory paper of the series, we set out a standard framework of research ethics with four ethical principles: respect for persons, beneficence, justice, and respect for communities [1]. Informed consent requirements for research participation stem from the principle of respect for persons, which requires that the wishes of autonomous individuals be respected, and that individuals with diminished autonomy be protected. Autonomous individuals are those who are capable of self-government and who can make responsible choices for themselves. Autonomous choices are decisions that are intentional, informed, and free of coercive influences [9].

The ethical principle of respect for persons may be viewed as deriving from deontological moral theory, which defines right action as the satisfaction of moral duties [10]. Kantian deontological theory posits that people have intrinsic moral worth by virtue of their capacity for rational decision making about their ends. Respect for the worth of others entails a moral duty to respect their autonomous choices. In other words, respecting the intrinsic moral worth of people requires that we treat them as ends in themselves, rather than as mere means to an end [10].

Informed consent may be viewed as a Kantian duty. Freedman explains informed consent as arising

"from the right which each of us possesses to be treated as a person, and in the duty which all of us have, to have respect for persons, to treat a person as such, and not as an object. For this entails that our capacities for personhood ought to be recognized by all -- these capacities including the capacity for rational decision and for action consequent upon rational decision" [11].

Here Freedman distinguishes how we may treat an object from how we may treat a person. An object -- be it a tennis ball or a computer keyboard -- does not have intrinsic moral worth; its worth is merely a function of its utility. Thus, objects are rightly used as mere means to our own ends, whether those ends be playing tennis or writing a paper. Freedman's point is that people may not be treated in this way. Each of us has a duty to "recognize the capacities for personhood" in others and demonstrate respect for it [11]. People are only legitimately involved in my project -- be it playing a game of tennis or coauthoring a paper -- when they agree to do so as a result of a rational decision. By providing informed consent, they in effect adopt my end as their own. In this way, I treat them not as mere means to an end, but as an end in themselves.

The central moral challenge of human subjects research is what legitimates putting a person at risk primarily or solely for the benefit of others. The purpose of human subjects research is the production of knowledge that will benefit others, be they future patients, the health system, or society as a whole. Although individual research subjects may in some cases experience direct benefits from study participation, they are in all cases put at risk to benefit others. Informed consent is a key part of the answer to this question. By providing informed consent to study participation, research subjects adopt the ends of the study as ends of their own. In obtaining informed consent, the researcher fulfills the duty to treat research subjects as persons and not objects, and as ends in themselves and not mere means to an end. In short, *the function of informed consent is to allow prospective research subjects to adopt the ends of the study as their own, thereby (partially) justifying exposing subjects to risk for the benefit of others*.

National and international research ethics guidelines lay out criteria for informed consent for research participation [12-15]. Disclosure requirements generally include the following elements: an explanation of the purpose of the study; a description of the study interventions; a description of the risks and potential benefits to subjects from research participation; a description of alternatives available to potential subjects should they choose not to participate; a description of confidentiality protections; a statement assuring potential subjects that participation is voluntary, that they may withdraw at any time, and that their quality of care will not be affected should they choose not to participate or to withdraw; and, information on whom they may contact with questions. As discussed in detail below, a number of these guidelines allow for a waiver of consent [12,13,15]. Accordingly, when a research ethics committee concludes that it is not feasible to obtain consent and study participation poses only minimal risk, it may approve a consent procedure which does not include, or which alters, some or all of these requirements, or it may waive the requirement to obtain consent.

## Addressing informed consent challenges in CRTs

In an individually randomized clinical trial (RCT), the human research subject is usually simultaneously the unit of randomization, the unit of experimentation, and the unit of observation [1]. Commonly, a patient is allocated randomly to receive one of two or more differing treatment regimens and data documenting the patient's response to the treatment are recorded. As a result, informed consent in RCTs tends to be straightforward. Informed consent is obtained from prospective research subjects prior to randomization and includes consent for random assignment, study interventions, and data collection.

Edwards and colleagues, Donner and Klar, and Hutton have observed that aspects of CRT design, including cluster randomization, cluster level interventions, and cluster size, present challenges to obtaining informed consent [2,3,16]. Clusters may be randomized before individual cluster members can be identified or approached. As a result, it is commonly not possible to obtain informed consent for randomization in CRTs. Cluster level interventions may be difficult for individual cluster members to avoid and, as a result, the refusal of informed consent may be meaningless. Further, when clusters are large it may not be feasible to obtain informed consent from all cluster members. Finally, the units of randomization, experimentation, and observation may differ so that one group may receive the experimental intervention while data are collected from a second group. This means that informed consent for random assignment, study interventions, and data collection may have to be obtained separately.

In what follows, we address key questions relating to informed consent and CRTs using the moral foundation outlined above and relevant national and international regulatory provisions.

### 1. How can a study proceed if informed consent is not possible?

As explained above, when a CRT involves a cluster level intervention or large cluster sizes, it may not be possible to obtain informed consent from study participants. A CRT reported by Rowland and colleagues illustrates well the practical difficulties in obtaining informed consent in certain cases [17].

Falciparum and vivax malaria are important health problems in Pakistan and indoor spraying of insecticide is the major preventive method used. The CRT sought to test the effectiveness of a new insecticide -- alphacypermethrin -- in controlling malaria rates in rural Pakistan [17]. The primary outcome measures were the annual incidence rates of falciparum and vivax malaria. The 180 km^2 ^study area in Punjab province was divided into nine sectors and each was randomized to spraying with one of two preparations of the insecticide or a no spraying control. In the two intervention arms of the study, all living quarters, storage rooms, and animal quarters were sprayed once with the insecticide. Survey teams visited 400 houses in each district every two weeks to identify new cases of malaria by symptom report and, when indicated, a blood smear to look microscopically for the parasite. Additionally, a cross-sectional survey collected blood smears from 200 to 300 school children in each sector before and after the intervention period. Village elders were informed of the study and gave their permission for the study to be conducted. The study concluded that the new insecticide reduced the annual incidence of falciparum malaria by 95% and vivax malaria by 80%.

In this CRT, all residents within the study area were deliberately intervened upon via manipulation of their environment and, hence, are human research subjects. However, it would have been impossible to obtain the informed consent of all research subjects in this study. As the study involved spraying all living quarters, storage rooms, and animal quarters within a geographic area, it would have been difficult for cluster members to avoid the intervention. Even if one refused to allow one's own home to be sprayed, one could not practically avoid all the treated buildings in the community (the insecticide proved to have an effective half-life of about six months). As a result, the refusal of informed consent in this study would have been meaningless. Second, requiring investigators to obtain the informed consent of research subjects would have rendered the study infeasible. Each of the nine study sectors contained approximately 2000 people living in 400 homes.

The practical impossibility of obtaining informed consent in such cases presents a serious ethical challenge to CRTs. Above we concluded that the function of informed consent is to allow prospective research subjects to adopt the ends of the study as their own, thereby (partially) justifying exposing subjects to risk for the benefit of others. Thus, informed consent plays a key role in the ethical justification of human subjects research. In the absence of some further justification, it seems that when informed consent cannot be obtained, a study cannot ethically proceed. Clearly, this conclusion has undesirable consequences; it would dramatically restrict the ability to conduct CRTs involving cluster level interventions or large clusters.

A partial solution is offered by provisions for a waiver of consent found in a number of national and international research ethics guidelines [12,13,15,18]. For instance, the Council of International Organizations of Medical Science *International Ethical Guidelines for Biomedical Research Involving Human Subjects *-- a widely acknowledged commentary on the *Declaration of Helsinki *-- contains the following provision:

"when the research design involves no more than minimal risk and a requirement of individual informed consent would make the conduct of the research impracticable (for example, where the research involves only excerpting data from subjects' records), the ethical review committee may waive some or all of the elements of informed consent" [15].

In short, a research ethics committee may grant a waiver of consent when it is not feasible to obtain consent and study participation poses only minimal risk. Limiting the use of a waiver of consent to minimal risk research is critical to its moral justification. Since informed consent is key to justifying exposing research subjects to risk for the benefit of others, waiving the informed consent requirement can only be contemplated in cases in which the risk involved is insignificant. When a study poses more than minimal risk, the lack of informed consent would amount to using research subjects as mere means to an end. Thus, when informed consent cannot be obtained and study procedures expose subjects to more than minimal risk, we believe a study cannot proceed ethically.

To illustrate how waiver of consent regulations may be applied in practice to CRTs, we examine the relevant provisions in the Common Rule, which governs all human subjects research supported or conducted by federal departments in the United States [12]. The Common Rule permits a research ethics committee to "approve a consent procedure which does not include, or which alters, some or all of the elements of informed consent set forth in this section, or waive the requirements to obtain informed consent provided the [research ethics committee] finds and documents that:

(a) The research involves no more than minimal risk to the subjects;

(b) The waiver or alteration will not adversely affect the rights and welfare of the subjects;

(c) The research could not practicably be carried out without the waiver or alteration; and,

(d) Whenever appropriate, the subjects will be provided with additional pertinent information after participation" [19].

Here we discuss each of these requirements.

#### (a) The research involves no more than minimal risk to the subjects

Minimal risk means that "the probability and magnitude of harm or discomfort anticipated in the research are not greater in and of themselves than those ordinarily encountered in daily life or during the performance of routine physical or psychological examinations or tests" [20]. In short, study interventions that pose minimal risk are commensurate with the risks of daily life. The risks of daily life are those that most or all of us -- often unthinkingly -- assume in the course of our daily lives [21]. These risks include a walk to the store for food and a routine visit to one's physician. In the context of research, routine healthcare, public health, or educational practice may be considered to involve only minimal risk. Also, certain methods of data collection, including interviews, surveys, medical records review, and physical examination, are commonly regarded as posing minimal risk [22].

Study interventions and data collection procedures in many CRTs in health research would fulfill the minimal risk criterion. Commonly, the study interventions evaluated are variations on routine care that do not pose additional risk to research subjects. Data collection commonly involves routinely collected medical information. Consider the malaria study described above [17]. In it, the study intervention involved the indoor spraying of one of two different preparations of an insecticide to control malaria. In Pakistan, the indoor spraying of insecticide is the primary approach for malaria prevention. The insecticide used in the study had undergone prior testing for safety and efficacy and was recommended for use by the World Health Organization [17]. Thus, the study intervention is consistent with standard public health practice in Pakistan; it poses no more risk than standard care and may be considered minimal risk. (The ethical analysis of benefits and harms in CRTs is the subject of another paper in this series.)

#### (b) The waiver or alteration will not adversely affect the rights and welfare of the subjects

This requirement is most helpfully viewed as complementing the requirement that the research involve no more than minimal risk. It requires that the use of the waiver of informed consent not violate any legal statute (U.S. states, for example California, may have specific informed consent requirements for research enshrined in legislation). Further, the research ethics committee must ensure that the welfare interests, including health interests, financial interests, and legal interests are not adversely affected by the waiver of consent. The fact that study participation poses only minimal risk provides a basis for the belief that the welfare interests of research subjects are sufficiently protected.

#### (c) The research could not practicably be carried out without the waiver or alteration

In CRTs in health research, there are broadly two circumstances in which a researcher might apply to a research ethics committee for a waiver of informed consent. In the first case, when a CRT involves a cluster level intervention or large cluster sizes, it may not be possible to obtain informed consent from study participants. In the second case, there may be concern that information disclosed during the consent process may substantially bias study outcomes or lead to selection bias. We discuss each case in turn.

In the first case, a waiver of informed consent may be sought because a refusal of consent would be meaningless or it is not feasible to obtain informed consent from all cluster members. No rule defines precisely in which circumstances "research could not practicably be carried out". Rather, this determination is within the discretion of the research ethics committee. The committee may consider a number of factors in making its judgment, including the size of the study group, the logistics of obtaining informed consent, and the financial cost involved [23]. It falls on the researcher to present a convincing case that these factors apply to a sufficient degree to the study under consideration to justify the waiver of consent.

The malaria study would likely satisfy the criterion that the study could not practicably be carried out without the waiver [17]. As we explained above, the study intervention could not reasonably be avoided by cluster members. With an average cluster size of about 2000 people in 400 homes, obtaining informed consent from all residents in each cluster would be difficult logistically. Therefore, a research ethics committee could reasonably conclude that the research could not be carried out without the waiver of consent.

It is important to note that a waiver of consent may apply to some, but not all, interventions in a CRT. This is most likely to occur in CRTs that involve both cluster level and individual level interventions [4]. Here again the malaria study is instructive. While the study intervention likely satisfies the requirements for a waiver of consent, interventions (in the broader sense of the term) for data collection in the study may not. Malaria annual incidence was ascertained by screening residents and obtaining blood samples of individuals with fevers and from a cross-section of school children [17]. It is generally feasible -- and therefore necessary -- to obtain informed consent for data collection procedures that involve interaction with research subjects, even if the procedures themselves pose only minimal risk. Thus, a waiver of consent would only apply to the spraying of insecticide in study communities, and researchers should have obtained informed consent for screening subjects and taking blood for peripheral smears.

Data collection procedures may be covered by a waiver of consent if it can be shown that the research would not be feasible without the waiver. Health services and knowledge translation CRTs commonly intervene on health professionals and measure patient outcomes. As we saw with the Guidelines Trial (discussed above), if the researcher does not obtain identifiable private information (or otherwise intervene upon or interact with patients), the patients are not research subjects and their informed consent is not required. However, if the study does involve the collection of identifiable private information, the researcher may apply for a waiver of consent for the patients in the study on the grounds that the research could not practicably be carried out otherwise [24].

In the second case, a waiver of consent application may be based on concern that information disclosed during the consent process may bias study outcomes or lead to selection bias [2,16,25,26]. CRTs commonly evaluate interventions aimed at modifying the behavior of cluster members. Knowledge of the nature of interventions in other arms of the trial, including the control arm, may at times plausibly be thought to change people's behaviors and consequently bias the outcome of the study (i.e., response bias). For example, consider a CRT evaluating an intervention to improve physician uptake of clinical practice guidelines. If physicians in the control group are informed of the details of the study intervention, they may change their behavior in accord with the practice guidelines, thus biasing the estimate of the intervention effect towards the null hypothesis [26]. In the worst case scenario, the study may conclude that an effective intervention is ineffective; on the other hand, if the bias is modest, the observed effect may still be of clinical interest and hence, influence policy.

Separate from response bias, selection bias can be introduced in a CRT when patients are required to provide informed consent for data collection after randomization of their health professionals [27]. Selection bias does not arise when all patients -- or a random subsample in each arm of the study -- consent; however, when there are different propensities to consent in the intervention and control arms of the study (e.g., when knowledge of, or exposure to, the experimental or control interventions influences the likelihood that patients consent to data collection) this can lead to imbalances between the trial arms which can bias the estimate of the intervention effect in an unknown direction and make the trial results uninterpretable. It has been argued that modifying or waiving informed consent requirements in these cases is justified on the grounds that it is unethical to undertake an experiment that is scientifically invalid [25].

Researchers can sometimes avoid or mitigate the risk of selection bias through careful planning of study procedures and execution [27]. For example, identification and recruitment can be completed before randomization if possible, or done by someone blind to the randomization status of the group. When such steps are inadequate, researchers may apply for a waiver of consent. There is no specific regulatory guidance defining when concerns for study validity might outweigh obligations to obtain informed consent. Generally, we believe that research ethics committees ought to adopt a restrictive stance on granting waivers of consent. When applying for a waiver of consent, investigators should provide evidence that complete disclosure would so bias the study findings as to make the study practically uninterpretable. When compelling evidence exists, the research ethics committee may approve an alteration of the consent procedures to ensure subjects are blinded to the exact nature of the interventions. Thus, these cases will involve an alteration of one or more of the disclosure requirements, rather than waiving altogether the requirement to obtain consent. Research ethics committees should not grant a waiver of consent in the absence of compelling evidence.

#### (d) Whenever appropriate, the subjects will be provided with additional pertinent information

Treating the research subject as a person, not simply as a means to an end, implies that researchers ought to make information available about the study whenever possible. Even when a waiver of consent has been granted on grounds that obtaining informed consent is not possible, it may nonetheless be feasible to inform cluster members of the existence of the study. Information may be provided through flyers, letters, or signs in healthcare institutions that a study is being conducted. Optionally, these communications may direct interested cluster members to a website or a contact person for further information.

### 2. Is consent to randomization always required?

In an individually randomized controlled trial, prospective subjects are approached for informed consent prior to randomization. In CRTs, however, it may not be possible to obtain informed consent until after randomization. Hutton observes that "[s]cientific and logistical constraints associated with [CRTs] imply that consent cannot necessarily be requested before an intervention is assigned to a person... In some cases it is logically impossible to obtain consent for the intervention prior to randomization of clusters" [16]. Even in CRTs involving an individual level intervention for which informed consent will be sought, random assignment of clusters may be completed before cluster members can be identified or approached. Some commentators have expressed the view that the inability to obtain consent for randomization may not respect subjects' autonomy rights [16,28].

The difficulty in obtaining informed consent prior to randomization is illustrated in a CRT described by Mullany and colleagues [29]. Infection of the umbilical stump (omphalitis) is an important cause of illness and death among newborns in developing countries. Mullany and colleagues conducted a CRT evaluating the effectiveness of an antiseptic -- chlorhexidine -- in preventing omphalitis. The study's primary outcome measures were infection of the umbilical stump and neonatal mortality. Clusters of 50-100 households, each served by a local health worker, were randomized to one of three interventions for newborns: swabbing the umbilical stump with chlorhexidine; cleansing it with soap and water; and dry stump care (the standard care control). From 2002 to 2005, women were approached for informed consent to study participation in the sixth month of pregnancy and baseline data on the household were collected. As random allocation of clusters occurred before the majority of women had become pregnant, it was impossible to obtain prior consent for randomization. The health worker visited the household within 24 hours of birth, when possible, and provided umbilical stump care on days 1, 2, 3, 4, 6, 8, and 10. On each occasion, any signs of infection were noted; questionnaires regarding neonatal care were completed on days 1 and 14. The study was approved by research ethics committees in Nepal and the United States. The study authors concluded that chlorhexidine reduced serious omphalitis by 75% and reduced the neonatal mortality risk by 24% compared with dry stump care [29]. There was no evidence that cleansing the umbilical stump with soap and water prevented infection.

The ethical principle of respect for persons generally requires that researchers obtain the informed consent of research subjects. We have said that the function of informed consent is to allow prospective research subjects to adopt the ends of the study as their own, thereby justifying exposing subjects to risk for the benefit of others. This implies that prospective research subjects should be approached for informed consent at the earliest opportunity and before they are exposed to risk. In patient RCTs, the earliest opportunity to approach potential subjects is prior to randomization. However, in CRTs it may not be possible to obtain individual informed consent until after randomization. Do CRTs in which consent to randomization cannot be obtained violate the ethical principle of respect for persons?

It is a dictum in ethics that "ought implies can": ethical obligations must be possible to fulfill. Given that it was impossible to approach prospective research subjects before randomization, researchers in this case approached women for informed consent to study participation when they were 6 months pregnant. The ethical question is whether obtaining the consent of women at this point achieves the moral purpose of informed consent. We believe it does. Women were approached well in advance of study or data collection interventions and, importantly, it is these interventions that place mother and child at risk (in part) for the benefit of others. They were informed of the purpose of the study, the interventions they would receive if they agreed to participate, and alternatives to study participation. Accordingly, the women approached for informed consent were free to adopt the ends of the study as ends of their own and to agree to participate in the study. Women who declined study participation avoided all study and data collection interventions and, thus, they were not exposed to risk for the benefit of others. We conclude that these consent procedures were sufficient to fulfill the moral purpose of informed consent.

As a general rule, prospective research subjects should be approached for informed consent at the earliest opportunity. In many CRTs, it is not possible to obtain informed consent prior to randomization of clusters. So long as prospective research subjects are approached for informed consent at the earliest opportunity, and prior to study or data collection interventions, we believe the ethical principle of respect for persons is satisfied. Although consent for randomization is not obtained, research subjects are nonetheless given the opportunity to adopt the ends of the study as their own before being exposed to risk for the benefit of others.

### 3. What information must be disclosed to potential subjects if their cluster has already been randomized?

Valid informed consent requires that prospective research subjects be given sufficient information to allow them to make a responsible decision regarding study participation [11]. As described above, national and international research ethics guidelines set out detailed requirements for informed consent [12-15,18]. The information that must be disclosed includes an explanation of the purpose of the study and a description of the study interventions. When informed consent takes place prior to randomization, prospective subjects are provided with detailed information on the study and data collection procedures in each of the trial's arms. Of course, at that time it is unknown to which of the trial's arms the subject will be allocated and, as a result, a responsible decision will require a detailed description of each of the possible future scenarios.

In the previous section, we discussed the ethical permissibility of approaching cluster members for informed consent after clusters have been randomized in a CRT. We concluded that if prospective research subjects are approached for informed consent at the earliest opportunity, and prior to study or data collection interventions, the moral purpose of informed consent is fulfilled. Here we ask what information must be disclosed to potential subjects if their cluster has already been randomized to a particular treatment arm? In these cases, must subjects receive a detailed description of study and data collection procedures in each of the CRT's arms?

When cluster members are approached for consent after clusters have been randomized, the informed consent process ought to be tailored to the study arm to which the cluster has been allocated. If prospective research subjects are to adopt the ends of the study as their own, the purpose of the study must be described in sufficient detail to allow them to decide whether the study's ends are consonant with their own values and priorities. Precisely what constitutes "sufficient detail" is a matter of judgment for the researcher and research ethics committee. At a minimum, a high-level description of the research question is required; we leave it open whether this description ought to name the other interventions compared in the study. Further, prospective subjects should be informed that their cluster has already been allocated to one of the study arms. To make a responsible decision regarding study participation, prospective subjects also need a clear understanding of the consequences of agreeing to or declining study participation. This means they must receive a detailed explanation of the study and data collection procedures in the trial arm to which their cluster has been allocated. But when the cluster has already been randomized to a particular study arm, the possible future scenarios entailed by other study arms have been foreclosed. Thus, while the study interventions *may *be named in the description of the study's purpose, a detailed discussion of the study and data collection procedures in other trial arms is not required. It is important to recognize that "tailored consent" is not a waiver of consent; rather, it is *full disclosure *in these circumstances as defined by the moral purpose of informed consent.

In the Nepal birth study, the informed consent document should have been tailored to the trial arm to which a particular cluster was allocated [29]. For example, the consent form provided to pregnant women whose cluster was assigned to the chlorhexidine arm should clearly describe the CRT's purpose. At a minimum, a high-level description of the research question is required, *viz*., "Infection of the stump of the umbilical cord is an important health concern for newborn babies. The purpose of this study is to determine the best means of cleaning the umbilical stump to reduce the risk of infection." Alternatively, a more detailed description of the CRT's purpose may be provided: "Infection of the stump of the umbilical cord is an important health concern for newborn babies. The purpose of this study is to determine whether cleansing the umbilical stump with a mild disinfectant, washing it with soap and water, or leaving it dry is the best means to reduce the risk of infection." The form should then explain that the cluster to which the woman belongs has already been allocated to one of the study arms, *viz*., "The health worker who will visit you and your baby has already been assigned to clean the umbilical stumps of babies with a mild disinfectant solution called chlorhexidine." The form should describe the chlorhexidine treatments and data collection procedures in detail. A detailed description of study procedures in other trial arms need not be provided.

One fortuitous effect of the use of a tailored informed consent in these circumstances is that the potential for bias may be mitigated. As we discussed above, commentators have expressed concern that information disclosed during the consent process may bias study outcomes [2,16,25,26]. While a clear description of the purpose of the CRT must be provided in all cases, when informed consent is tailored to the cluster's assignment, *detailed *information on study procedures in other trial arms need not be provided. This may reduce the chance that detailed information on study procedures in other trial arms will change the behavior of the research subject. It should be noted, however, that concerns about selection bias are not affected by this proposal.

### 4. Is passive consent a valid substitute for informed consent?

In CRTs of cluster level interventions some researchers have used so-called "passive consent" to recruit research subjects. In passive consent, information about the CRT is provided to prospective research subjects or their surrogate decision makers [26,30-32]. If the prospective subject does not object to being enrolled in the CRT, then the subject is presumed to have agreed to participate. Here we ask whether passive consent is an acceptable approach to obtaining the informed consent of research subjects.

Passive consent is sometimes used in CRTs in education and involves sending information about the study home with students. If a student does not return a document signed by a parent declining study participation, the parent is presumed to have consented on behalf of the student [32]. The information sent home with the student often contains sufficient information about the study to enable parents to make an informed decision regarding study participation [32]. Nonetheless, there are several shortcomings to passive consent in this setting. The researcher cannot know whether children actually provided the study information to their parents. Further, even if the information is provided to a parent, there is no assurance that the parent is capable of understanding the information provided [32]. Thus, the absence of a written refusal of study participation may equally reflect valid informed consent, invalid consent based on misunderstanding, or lack of consent because the parent never received the information.

In health services research, passive consent is used somewhat differently. In CRTs conducted in healthcare settings, notices may be posted in patient areas that research is being conducted, although detailed information about the study is usually not provided. The notices indicate that if the patient does not wish to participate in the study, then the patient may contact the investigator to opt out [26,31,32]. While notices may serve to increase awareness about the research, passive consent in this setting also has shortcomings. Typically, notices do not provide sufficient information to allow for an informed decision. Further, the absence of contact from a patient regarding the study may equally reflect a desire to participate in the study, or a failure to see the notice.

As discussed above, the function of informed consent is to allow prospective research subjects to adopt the ends of the study as their own, thereby justifying exposing subjects to risk for the benefit of others. Given the shortcomings of passive consent, it is difficult to see how it can reasonably fulfill the moral function of informed consent. Passive consent is better conceived of as an alteration of consent; as such, it is subject to the same regulatory demands as a waiver of consent [12,13,15,18]. The researcher who wishes to use passive consent must convince the research ethics committee that the study is not feasible without the alteration of consent and study participation poses only minimal risk.

Passive consent is not any more protective of research subjects' autonomy rights than a waiver of consent. Its use may, however, be justified on pragmatic grounds. For example, in school-based CRTs, passive consent may be required by school administrators or parent groups [30]. Other considerations may prompt the use of passive consent in health care settings. For instance, a hospital-based CRT evaluating a quality improvement intervention for diabetes care used passive consent because "clinicians and the laboratories owned by community hospitals were worried about public concern as patients discovered they were enrolled without consent into a research project" [30].

### 5. Do health professionals have a moral obligation to participate as subjects in CRTs designed to improve professional practice?

Knowledge translation studies commonly intervene on physicians and other health care professionals to change practice. For instance, in the Guidelines Trial (discussed above), birth attendants in hospitals randomized to the intervention arm were trained in guidelines for the use of episiotomy and the management of the third stage of labor and received reminders regarding key practices [8]. Since the birth attendants were the direct recipients of the study intervention, they were human research subjects. Researchers have a general obligation to obtain the informed consent of research subjects. In the Guidelines Trial, researchers obtained the informed consent of all birth attendants in the study. However, in cases in which the intervention is administered to groups of health care providers or if their numbers are large, it may not always be possible to obtain informed consent. In these circumstances, researchers may apply to the research ethics committee for a waiver of consent.

Some have argued that health care professionals have an obligation to participate in health systems or knowledge translation CRTs [16,25]. Hutton argues that "[i]n some cases, the experimental units, professionals, might have a duty to enroll as part of their continuing professional development" [16]. In a subsequent article, Hutton and colleagues make the following further claim: "[I]f a health care professional chooses not to participate in a study, they are in effect denying their patients the potential benefits of participation. Health care providers ought to do the best for their patients..." [25]. Here we ask whether continuing professional development or the prospect of benefit for patients ground an obligation for health professional to participate as research subjects in CRTs. If they do, does this imply that the informed consent of health professionals is not required?

Health care professionals are required to engage in continuing professional development as a condition of licensure in many jurisdictions [33]. Physicians are afforded a great deal of latitude in determining the means by which continuing education is obtained. Acceptable options may include self study, conference attendance, teaching preparation, and participation in educational programs leading to a diploma or degree [34]. The wide discretion in choosing professional development activities undermines the claim that health professionals have an obligation to participate as subjects in a CRT. The simple fact is that health care professionals may achieve the end of improving their skills and knowledge through other -- equally legitimate -- means.

Regarding the second argument, it is true that knowledge translation and quality improvement studies seek to improve patient care. But the fact that an educational or quality improvement intervention is being evaluated in a CRT suggests that its effectiveness is unproven. Indeed, if it was known at the start of the trial that the study intervention is effective, the CRT would be unethical [35]. Additionally, the claim that the health care professional has an obligation to participate in research seems difficult to square with the fact that such CRTs will include a usual care or no intervention control group. If denying patients access to the benefit of the study intervention is morally questionable, then how is the control arm ethically justified? (The applicability of clinical equipoise to questions such as these in CRTs is explored in another paper in the series [36]). Thus, the prospect of denying patients potential benefits does not seem to ground an obligation for participation.

A more fruitful line of reasoning starts with the recognition that health research is a socially important activity. Such research is critical to ensure that treatments are safe and effective, and that new treatments for disease continue to be developed. Health services and knowledge translation research help ensure that when advances are made, they are actually implemented in medical practice in sustainable and affordable ways. It has been argued that the social importance of health research grounds a general obligation to support and even participate in research [37]. This may be thought to apply equally to patients and health care professionals. Thus, as Hutton and colleagues put it, "[t]his might suggest that [health care professionals] should have a high threshold for opting out of implementation research studies" [25]. In other words, health professionals ought to have a good reason to decline participation as subjects in a CRT. It is important to recognize that our position falls well short of concluding that a health professional has an enforceable obligation to participate in a specific trial (as one could always participate legitimately in another study) [37]. Finally, recognizing this general obligation does not diminish -- in any way-- the need to obtain the informed consent of health care professionals [37].

## Conclusion

This paper addresses the question: from whom, when, and how must informed consent be obtained in CRTs in health research? The ethical principle of respect for persons implies that researchers are generally obligated to obtain the informed consent of research subjects. Aspects of CRT design, including cluster randomization, cluster level interventions, and cluster size, present challenges to obtaining informed consent. Here we address five questions related to consent and CRTs: How can a study proceed if informed consent is not possible? Is consent to randomization always required? What information must be disclosed to potential subjects if their cluster has already been randomized? Is passive consent a valid substitute for informed consent? Do health professionals have a moral obligation to participate as subjects in CRTs designed to improve professional practice?

We set out a framework based on the moral foundations of informed consent and international regulatory provisions to address each of these questions. First, when informed consent is not possible, a study may proceed if a research ethics committee is satisfied that conditions for a waiver of consent are satisfied. Second, informed consent to randomization may not be required if it is not possible to approach subjects at the time of randomization. Third, when potential subjects are approached after cluster randomization, they must be provided with a detailed description of the interventions in the trial arm to which their cluster has been randomized; detailed information on interventions in other trial arms need not be provided. Fourth, while passive consent may serve a variety of practical ends, it is not a substitute for valid informed consent. Fifth, while health professionals may indeed have a moral obligation to participate as subjects in research, this does not diminish the necessity of informed consent to study participation.

## Note

We have created a Wiki webpage to facilitate an open discussion about the ideas expressed in this and other papers published in the series on ethical challenges in CRTs. Please enter your thoughts and comments at http://crtethics.wikispaces.com.

## Competing interests

AB, JCB, ADM, RS, MT, CW, and AW: None declared

RB, AD, MPE, and JMG have all submitted cluster trial protocols to ethics committees and had difficulty explaining to them the differences between cluster randomized trials and individual patient clinical trials.

## Authors' contributions

ADM, CW, AB, MT and JMG contributed to the conception and design of the manuscript.

ADM wrote the initial draft, and ADM and CW led writing of subsequent versions.

All authors commented on sequential drafts and approved the final version.
